# Barriers to access to visceral leishmaniasis diagnosis and care among seasonal mobile workers in Western Tigray, Northern Ethiopia: A qualitative study

**DOI:** 10.1371/journal.pntd.0006778

**Published:** 2018-11-08

**Authors:** Rebecca Marie Coulborn, Tesfay Gebregzabher Gebrehiwot, Martin Schneider, Sibylle Gerstl, Cherinet Adera, Mercè Herrero, Klaudia Porten, Margriet den Boer, Koert Ritmeijer, Jorge Alvar, Abrahim Hassen, Afework Mulugeta

**Affiliations:** 1 Epicentre, Paris, France; 2 School of Public Health, Mekelle University, Mekelle, Ethiopia; 3 KalaCORE, London, United Kingdom; 4 World Health Organization, Geneva, Switzerland; 5 Médecins Sans Frontières, London, United Kingdom; 6 Médecins Sans Frontières, Amsterdam, The Netherlands; 7 Drugs for Neglected Diseases Initiative, Geneva, Switzerland; 8 Department of Health Promotion and Disease Prevention, Tigray Regional Health Bureau, Tigray, Ethiopia; Liverpool School of Tropical Medicine, UNITED KINGDOM

## Abstract

**Background:**

Ethiopia bears a high burden of visceral leishmaniasis (VL). Early access to VL diagnosis and care improves clinical prognosis and reduces transmission from infected humans; however, significant obstacles exist. The approximate 250,000 seasonal mobile workers (MW) employed annually in northwestern Ethiopia may be particularly disadvantaged and at risk of VL acquisition and death. Our study aimed to assess barriers, and recommend interventions to increase access, to VL diagnosis and care among MWs.

**Methodology/Principal findings:**

In 2017, 50 interviews and 11 focus group discussions were conducted with MWs, mobile residents, VL patients and caretakers, community leaders and healthcare workers in Kafta Humera District, Tigray. Participants reported high vulnerability to VL among MWs and residents engaged in transitory work. Multiple visits to health facilities were consistently needed to access VL diagnosis. Inadequate healthcare worker training, diagnostic test kit unavailability at the primary healthcare level, lack of VL awareness, insufficient finances for care-seeking and prioritization of income-generating activities were significant barriers to diagnosis and care. Social (decision-making and financial) support strongly and positively influenced care-seeking; workers unable to receive salary advances, compensation for partial work, or peer assistance for contract completion were particularly disadvantaged. Participants recommended the government/stakeholders intervene to ensure: MWs access to bed-nets, food, shelter, water, and healthcare at farms or sick leave; decentralization of diagnostic tests to primary healthcare facilities; surplus medications/staff during the peak season; improved referral/feedback/reporting/training within the health system; free comprehensive healthcare for all VL-related services; and community health education.

**Conclusions/Significance:**

Contrary to what health policy for VL dictates in this endemic setting, study participants reported very poor access to diagnosis and, consequently, significantly delayed access to treatment. Interventions tailored to the socio-economic and health needs of MWs (and other persons suffering from VL) are urgently needed to reduce health disparities and the VL burden.

## Author summary

Ethiopia bears a high burden of visceral leishmaniasis (VL)—a neglected tropical disease transmitted through the bite of a sand fly that disproportionately affects vulnerable populations. Without treatment, VL progresses, causing increasingly severe symptoms and ultimately death within two years, in most cases. Early access to VL diagnosis and care improves clinical prognosis and reduces transmission from infected humans; however, significant obstacles exist. To our knowledge, our study is the first in Ethiopia to use qualitative methods to assess barriers to VL diagnosis and care among seasonal mobile workers. Strikingly, we found that contrary to what health policy for VL dictates in this endemic setting, study participants reported very poor access to diagnosis and, consequently, significantly delayed access to treatment. Our findings have important public health and policy implications. Specifically, our results offer strategies that may increase access to VL diagnosis and care, allowing for earlier treatment and better prognoses for VL patients, and reducing the potential health disparities and the overall burden of VL.

## Introduction

### Context

Visceral leishmaniasis (VL) is a neglected tropical disease caused by the protozoan *Leishmania donovani* in the Indian subcontinent and East Africa, and *Leishmania infantum* in Latin America and the Mediterranean basin [1,2]. The bite of the sand fly transmits VL [2]. The World Health Organization (WHO), using reported cases from 2004 to 2008, estimates 188,900 to 365,500 new VL cases each year in the six high VL burden countries of Bangladesh, Ethiopia, India, Nepal, South Sudan and Sudan [3]; newer estimates (based on reported cases from 2010–2014 in the same six countries) range from approximately 41,000 to 68,000 new VL cases annually (Dr. Josè Antonio Ruiz-Postigo, oral communication at the 6th World Congress on Leishmaniasis, Toledo, Spain, May 17, 2017). These six countries, together with Brazil, account for more than 90% of the worldwide VL cases [2].

In Ethiopia, VL is primarily anthroponotic [4]. The estimated incidence of symptomatic VL in Ethiopia (based on reported cases 2004–2008) ranges from 3,700 to 7,400 cases per year, with two- to four-fold underreporting surmised [3]; newer estimates (based on reported cases 2010–2014) range from approximately 2,600 to 3,900 cases per year, with an estimated underreporting factor of 1.2 to 1.8 (Dr. Josè Antonio Ruiz-Postigo, oral communication at the 6th World Congress on Leishmaniasis, Toledo, Spain, May 17, 2017). Endemic areas include the northwest, southwest, central and southern lowlands, with the northwest region bearing the greatest burden of disease [3]. Seasonal dynamics exist; environmental and climatic conditions that influence the distribution and abundance of sand fly vectors are associated with peak periods of VL transmission in human populations [5–7]. Furthermore, drug-resistant strains, immune suppression linked to malnutrition and HIV co-infection, and population movements contribute to VL emergence and re-emergence [8,9]. In the VL endemic Humera-Abdurafi-Metema area of Ethiopia, agricultural development has spurred the migration of approximately 250,000 mobile workers (MWs) each year [10]. This surge in economically driven migration corresponds to increases in the number of VL cases, and associated deaths, in areas that previously did not experience VL epidemics [3,11].

Without treatment, VL is almost always fatal [12]. Delays in detection and treatment increase the risk of morbidity and mortality as well as the dissemination of disease to others [12]. Accordingly, early access to VL care is imperative to improve clinical prognosis and reduce transmission via human reservoirs. Unfortunately, significant barriers to access to care exist.

In Ethiopia, known obstacles to access to VL care include a suboptimal number of health facilities offering diagnosis and treatment as well as transportation constraints [3]. Insufficient financial resources for care-seeking also hinder access; though VL diagnostics and treatment are free in the country, patients must pay other healthcare fees and associated costs [3]. VL awareness, among certain populations and healthcare providers, is also limited [3,11,13]. To address these challenges, numerous partners, including WHO, in collaboration with the Ethiopian Federal Ministry of Health, support VL research and healthcare service delivery [14]. At present, Médecins Sans Frontières (MSF) offers comprehensive free care for VL patients at a hospital in Abdurafi [14]. In addition, KalaCORE, a consortium of partners including Drugs for Neglected Diseases Initiative, the London School of Hygiene and Tropical Medicine, Mott MacDonald Ltd and MSF, is supporting the expansion of diagnostic and treatment services for VL through clinical mentoring, supply provision, and other interventions [14]. Currently in Ethiopia, diagnostic test kits and treatment for VL are primarily available at hospitals; very few health centres offer VL diagnostics and even fewer offer VL treatment. Despite progress to expand capacity, access to VL diagnosis and care remains problematic, particularly among certain populations.

Previous research has found intense transmission of VL among MWs, who may be disproportionately affected by barriers to access to care, and subsequently at greater risk of VL acquisition and death [11,15,16]. Reported factors associated with an increased likelihood of clinical manifestations of VL and poor access to diagnosis, management, and care of VL cases, include: movement from non-endemic areas (where individuals have no/low VL immunity) to VL endemic areas; poor living conditions and lack of adequate health care provision; low socio-economic status [9,17,18]; and high HIV-prevalence rates [19,20]. In addition, poor nutritional status, characteristic of MWs, is thought to be important although not formally proven [21]. Given that MWs also return home, often moving from endemic to non-endemic VL areas, they may be more likely to encounter health workers who lack prior experience with VL [11,13]. During the 2004 to 2007 VL epidemic in Libo Kemkem, healthcare workers mistook early cases of VL among returning MWs for drug-resistant malaria. Data suggest at least a 2-year gap from symptom onset to diagnosis by the time some VL cases were properly identified [11,13]. Such impediments have significant consequences at both the individual and population level, such as increased transmission of disease and economic consequences associated with absence from work and healthcare costs. Understanding the extent of barriers to VL care among mobile populations is essential in order to facilitate interventions to overcome potential health disparities and reduce the overall burden of VL. However, current knowledge is lacking.

### Study aim

To address gaps in knowledge, our study aimed to assess obstacles to VL diagnosis and care, and ultimately to provide recommendations to guide the development of innovative strategies to increase access to VL care among MWs in Kafta Humera District, Tigray, Ethiopia.

## Methods

### Ethics statement

The Health Research Ethics Review Committee of the College of Health Sciences of Mekelle University in Ethiopia (Reference: ERC 0796/2016) and the Comité de Protection des Personnes Ile de France XI in France (Reference: 16036) approved the study. Study implementation observed the principles of the Declaration of Helsinki and the International Ethical Guidelines for Biomedical Research involving human subjects, published by the Council for International Organizations of Medical Sciences. All participants provided written informed consent.

### Setting

The study took place in the highly VL endemic lowland area of northwest Ethiopia, near the town of Humera, Tigray National Regional State. Large-scale agriculture plays an important role in the local economy. Production of sesame, cotton and sorghum annually attracts many MWs from other areas of Tigray and various regions in Ethiopia. MWs stay in the farm areas near Humera for months during the agricultural season.

### Study design

The study used a qualitative research design comprised of focus group discussions (FGD) (aiming to include six to ten participants per group) and semi-structured, in-depth interviews as the main data collection techniques. A topic guide helped to ensure that interviewers addressed the main themes of the study, but did not limit deeper exploration of emergent themes that arose during the interviews and FGDs [22]. This approach enabled the exploration of the opinions, beliefs, feelings and needs of the study population as they relate to VL diagnosis and treatment [22].

### Definitions

Table 1 presents the definitions used in the study, based, in part, on those in previously published literature [10,23,24].

**Table 1:** Study definitions used for participant categories.

Mobile worker	Individual who spent <6 months in the study area over the past year, with their principal residence outside of the study area, who engaged in agricultural work for hire on property belonging to someone else
Mobile resident	Individual who spent ≥6 months in the study area over the past year, with their principal residence in the study area, but who nevertheless engaged in agricultural work for hire on property belonging to someone else
Caretaker of VL patient	Friend or relative who assisted a former or current VL patient during the course of their illness and/or stay in the hospital (at the time of the study or in the past)
VL patient	Mobile worker, resident, or mobile resident who received healthcare (at the time of the study or in the past) from a health facility for VL in the study area
Community leader	Resident who had a leadership role within the community (i.e., religious leader, civic leader, political leader) of the study area
Healthcare worker	Clinical staff member employed at a public or private health facility in the catchment area

### Participant recruitment

Males and females, at least 18 years of age, were recruited using purposive and snowball sampling techniques. Six different categories of study participants were included: 1.) Healthy MWs, 2.) Healthy MRs, 3.) Caretakers of VL patients, 4.) Current and former VL patients, 5.) Community leaders, and 6.) Healthcare workers. New participants were recruited until no relevant new information was provided from additional interviews (i.e., the point of saturation was reached) [25,26].

Current patients were identified in the VL ward of Kahsay Abera Hospital, the district hospital in Humera (and only hospital in Kafta Humera providing VL treatment). Former patients—those completing VL treatment in 2015, 2016 or 2017—and caretakers were identified based on reviews of health records at health facilities and discussions with healthcare workers, current patients, and authorities; they were traced and recruited from the villages of Adebay, Baeker, Bereket, and Maykadra. MWs and MRs were recruited at the meeting points in Humera and Maykadra where laborers gather in search of work. Community leaders were contacted at their offices or places of activity. Healthcare workers were identified in Kahsay Abera Hospital and in the health centres of Baeker, Bereket, and Maykadra. The field coordinator of the study, clinical staff, health extension workers, and staff from Tigray Regional Health Bureau assisted with participant recruitment. All participants were contacted one to two days prior to interviews or FGDs.

### Data collection

Eight academic staff from the University of Mekelle, Ethiopia, were recruited for data collection. They received training on principles of qualitative research with a special emphasis on methods for conducting interviews and animating FGDs and on the epidemiology, diagnosis, and treatment of VL. In addition, with patients and health staff from Shire Hospital (Tigray National Regional State), the research team piloted the interview/FGD topic guide, to ensure its comprehensibility and potential to elicit discussion, while also honing their interviewing skills. The topic guide was adapted based on the results of the pilot. It was also modified following reviews of the interview/FGD transcripts and supervision sessions with senior researchers during data collection for the identification of themes or points that required more detailed or new questioning in subsequent interviews.

The topic guide included the following core themes: 1.) Migration of MWs, 2.) Perception of illness & knowledge of VL, 3.) Sources of information on VL, 4.) History of VL illness and health-seeking behavior, 5.) Satisfaction with healthcare providers and the medical system, 6.) Adherence to VL treatment, 7.) Beliefs and preferences for healing/healthcare services, 8.) Accessibility and barriers to VL healing/healthcare services, 9.) Decision-making within the family/community for choosing to pay for or seek external care, 10.) Perception of the VL economic burden, 11.) Practices and perceptions of healthcare providers, and 12.) Perception of previous interventions and suggestions of changes/future interventions.

Interviews and FGDs were recorded using digital recorders. Sex, age, residence, education level and occupation were collected to have a general idea of the study population and to categorize participants; this information was written down on paper during the interview and later entered into a spreadsheet. Care was taken to find a location that allowed for confidentiality and suitable sound recording quality. The interviews/FGDs took place in the health centres or the hospital, at the homes of participants, or on the premises of the individual’s professional environment. Interviews were conducted with one data collector. For FGDs, data collectors worked in pairs, with one serving as the moderator, and the other as the note taker. Each data collector worked in each role, according to the senior researchers’ plan. All interviews/FGDs were conducted in the local language with which the interviewees felt most comfortable; most interviews were in Tigrigna, and a few were in Amharic. At the completion of the interview, each participant was informed about and received compensation in Ethiopian Birr for their participation. The compensation was in accordance with the principles of the Declaration of Helsinki and the International Ethical Guidelines for Biomedical Research involving human subjects to compensate for loss of income during study participation. Daily debriefing sessions occurred between the senior researchers and data collectors to ensure quality assurance, review of identified themes, and strong communication.

### Data transcription, translation and analyses

After each recording, data collectors transferred audio-files to a computer. Data were transcribed and then translated into English. The senior researchers reviewed all English translations, ensuring comprehensibility without specific local knowledge. Uncertainties and ambiguities were discussed with the data collectors and reformulated to a common agreement.

Data were imported into Atlas.ti (Version 7.0, Berlin) qualitative data analysis software. Three researchers (the principal investigator and two senior researchers) coded the data using inductive qualitative content analysis [27]. Emergent categories and themes were identified based on meticulous and systematic reading and coding of the transcripts. Codes and sub-codes were refined. Each transcript was coded, at minimum, by one researcher and reviewed, at minimum, by one other researcher. The principal investigator reviewed all coding and ensured cohesion in the approach and use of themes.

All transcription, translation, and analyses occurred with anonymized data.

## Results

### Characteristics of the study population

From 28 January to 23 February 2017, 50 interviews and 11 FGDs were conducted, totaling 137 study participants. Table 2 presents the number of FGD and interview sessions conducted according to each participant category.

**Table 2:** FGD and interview sessions according to participant category.

Participant category	Number of sessions conducted	
	Interview	FGD
Mobile worker	6	2
Mobile resident	0	1
Caretaker of VL patient	9	2
VL patient	10	3
Community leader	11	0
Healthcare worker	14	3
**Total**	**50**	**11**

FGD = Focus group discussion; VL = visceral leishmaniasis.

Interviews lasted, on average, 45 minutes. Among interview participants, the median age was 31 years (range: 19–71 years) and 76% were male. Most participants originated from Kafta Humera District; only one participant originated from a highland area of Ethiopia. Nearly one quarter of interview participants had either no formal education or only the ability to read and write. Occupations of interview participants included: religious leaders (i.e., imam, nun, priest), political and public health authorities, civic authorities/leaders, hired farmers, subsistence-level farmers, farm owners, herders, tractor drivers, medical professionals (e.g., doctor, nurse, pharmacist), traditional healers, and students.

FGDs ranged in size from six to 10 participants—approximately 60% of whom were male—and lasted on average 94 minutes. The following occupations were represented: hired farmers, subsistence-level farmers, medical professionals (i.e., pharmacy technician, nurse, health extension worker, public health nurse), heads of health centers, wives of hired farmers, students and drivers.

S1 and S2 Tables provide in more detail the characteristics of participants included in the interviews and FGDs, respectively.

### MW routines and behavior shaped by work intensity on the farms

#### Migration

MWs came from all over Ethiopia, particularly from the Tigray and Amhara States, in pursuit of agricultural work. In addition, some local residents, either because of their residence in a very remote area or out of economic necessity, also engaged in mobile seasonal work (i.e., mobile residents (MR)).

“I was going to the farmland for daily working. Since I am poor, I have been working with the farm owners as a daily worker.”

Interview, Caretaker of VL patient.

Crops influenced MW movements. MWs reported the period from February to May as the time for clearing and preparing the farmland, with few workers present. Conversely, the weeding season, which starts in June, ushers in a massive influx of mobile seasonal workers.

“…at least one thousand MWs are engaged in farm work under a single farm owner”

Interview, MW.

MWs reported that most stayed through August/September, during the rainy season. Some remained even longer to participate in harvesting (or went home first and then returned for harvesting), staying through early November for sesame, December for sorghum, and even later for cotton. The time spent in the area of Humera for MWs ranged from weeks to months, with larger farms retaining workers for longer. Additionally, MWs with families at home and students restarting classes returned home sooner than other individuals.

#### Work schedule

MWs usually worked eight to 18 hours a day, often at night and avoiding the hottest hours of the day. Some worked as day laborers, others under contract, with the latter based on work produced rather than a fixed number of hours/days. Contracted MWs who worked quickly could complete their jobs sooner and move more rapidly to the next contract; the intensity of work meant that MWs skipped meals and took only short naps.

#### Sleeping place

MWs generally slept on the farmlands, in the area where they were harvesting, directly on the ground (under trees or on termite hills), on the straw of the crops, on a sack, on local beds made from stone and mud or using a sort of tent. While some camps had houses where MWs could sleep, they were not always available, because either the work area was too far away from the houses or the farm owner did not offer the worker this option. Bed-nets were either not used, or used only during the cool season.

#### Eating habits

For food preparation, MWs were not supposed to move from their work place, but rather to cook their food on the farm, using the materials provided by farm owners. MWs prepared porridge from ground sorghum, salt, and pepper supplied from farm owners. Many MWs described the provision of unclean and unsafe food. Some even stated that during supervisory visits by government officials to the camps, farm owners attempted to conceal spoilage, only to later distribute the spoiled food to MWs.

“There are delegates [who] came from the district office to supervise the camps. Even though the law says so, in most of the camps, there is no health service, no house for sleeping and no safe food or water supply. Meaning, the workers, including the permanent employees, will not get these services. It is a very challenging life. For example, I was working in one of the biggest farms as a supervisor. One day the district delegates came to the farm and in the store, there was completely spoiled flour. He told us to remove and discard it. However, since the security worker was a relative of the farm owner, he told me not to discard but to put it behind the store. Then, the security had left the camp; the security worker made a telephone call to the farm owner and ordered us to return and to distribute the spoilt flour to the MWs. The farm owner told us, ‘no one has the right to decide on my property. No one has the right to interfere with my business,’ he said. Then, because of the consumption of the spoilt flour by the workers including me, we faced a lot of problems.”

FGD, MW.

### Perceived vulnerable groups

Participants from all respondent categories stated that sleeping around black cotton soil, under trees or near sandy areas, living around farming areas in the lowlands, hunger, and fatigue put individuals at risk of VL. In addition, individuals without adequate financial resources, those belonging to younger age groups, those lacking knowledge and experience with VL, those not wearing protective long-sleeved clothes, individuals without family nearby, and those not using bed-nets were considered particularly at risk.

The aforementioned risk factors characterized MWs, whom study participants from all respondent categories consistently named as the most vulnerable group for VL. Residents engaged in agricultural work, particularly mobile residents (MRs), were also considered vulnerable.

“…we MWs are highly at risk.”

FGD, MW.

“Even herdsmen are at a high risk of getting the disease VL as they spend longer period of time around farming areas.”

IDI, VL patient.

### Divergent levels of community awareness of VL and limited health education

Different levels of community awareness were reported. Considering all respondent categories, many participants felt that community awareness of VL was high, while others stated that it was low.

The divergence in opinions appeared related to the population in question, with MWs much less aware than the resident population. Nevertheless, even for those with greater knowledge, awareness alone was insufficient for early access to diagnosis and care.

“Communities near Humera are aware of the presence of the disease. However, patients did not come early [to healthcare] despite their information about the disease.”

Interview, Healthcare worker.

Most community members (i.e., MWs, MRs, caretakers of VL patients, VL patients) and community leaders correctly named the lowland areas of Western Tigray as an area endemic for VL. They also largely attributed VL transmission to the bite of a sand fly and reported cracked black cotton soil as well as the area under balanite and acacia trees as the sand fly habitat.

“…fly-like animals which are mostly found in desert areas like this place where we are working. These insects bite us in the night. It is through these flies that VL is transmitted.”

Interview, MW.

However, community members and leaders often simultaneously proposed other modes of transmission, including: contact with a leaf; contaminated food; dirt/sand/lack of sanitation; animal dung; evil; contact with a fomite; increased body temperature; the bite of a mosquito; person-to-person transmission, unclean/stagnant water; or worms.

“… [VL is] caused by lack of sanitation and some people say that it is caused by contact with a leaf, but I do not know whether it is true or not.”

Interview, Community leader.

Furthermore, numerous community members and leaders attributed VL to a process of ‘disease evolution’, whereby malaria evolved into VL directly, or via an intermediary step(s) including typhoid fever and/or pneumonia; some stated this resulted from not seeking treatment early.

“Malaria is converted to typhoid fever…and finally the disease becomes VL.”

Interview, Community leader.

“If they get the treatment [for malaria] early, they will not develop VL. But if they do not get the treatment, they will develop [VL]”

Interview, MW.

Considering all respondent categories, some participants considered VL health education adequate, while the vast majority found it insufficient. That health education was largely facility-based rather than community-based represented the main criticism.

“We could not go down to the level of the ordinary public for awareness creation. Our effort to do that is low. The only thing we do is give information to admitted patients and their caregivers during admission, care, and discharge.”

Interview, Healthcare worker.

Even when reaching the community, health education was either void of information on VL, too general, or delivered inconsistently.

“We don’t have health professionals that provide health education about VL in our kebelle [district]. When we take health professionals to our farm area, they simply tell us to provide our daily workers with clean water for drinking. They do not tell us what we should do and support the MWs when they sleep. Moreover, we ourselves don’t teach them.”

Interview, Community leader.

Local community groups, capable of informing and mobilizing the community, were considered underutilized. Patients previously treated for VL constituted the main source of VL information for the community.

### Perceived mechanisms of VL prevention

Community members and leaders offered many suggestions for preventing VL, linked to the various modes of transmission they attributed to VL acquisition. Table 3 presents the protective measures mentioned by community members and leaders, categorized according to their focus on the environment or person.

**Table 3:** VL protective measures according to community members and leaders.

**Environment-focused**
Living in a windy area
Avoiding standing water
Avoiding or clearing certain trees
Filling in cracked loam/soil
Maintaining environmental hygiene
Avoiding working midday/during powerful sun
Not sleeping on the ground
Sleeping in a bed
Sleeping on an elevated surface
Sleeping with a blanket or sack covering the body
Sleeping under a bed-net
**Person-focused**
Using insecticides or ointments (i.e., Vaseline) on the body
Going early to the health center
Getting adequate rest
Eating good food
Drinking clean water
Maintaining personal hygiene

Among the suggested protective measures, bed-nets constituted the measure mentioned the most often as an important VL prevention strategy.

“During the night, they [sand flies] are active and usually they are blown away if there is wind. However, if conditions are not windy, they [sand flies] will not be blown away and they [sand flies] bite us. We spend the whole nighttime scratching our body. Therefore, we need to prevent them [sand flies] by using bed-nets.”

Interview, MW.

Nevertheless, across all respondent categories—including MWs themselves—participants repeatedly and consistently stated that MWs did not use bed-nets. Reports of use of bed-nets by residents were inconsistent, with some participants stating that residents always used bed-nets and others stating the opposite.

Participants provided many explanations for the non-use of bed-nets. Table 4 presents these reasons, categorized according to financial/procurement and utility/credibility constraints.

**Table 4:** Reasons for non-use of bed nets.

**Financial or procurement constraints**
Financial expense associated with bed-net purchase
Inability to find bed-nets at farms (i.e., bed-net purchase had to be made prior to coming to farms)
**Utility or credibility constraints**
Bed-net trapped heat during the high temperatures of the hot season
Bed-net ill-suited for MW sleeping situation (i.e., for “indoor use”, whereas most MWs slept outside)
Doubts regarding bed-net effectiveness due to the size of their mesh

Regarding one of the constraints, that of the effectiveness of bed-nets in relation to mesh size, participants perceived that the width of the mesh of bed-nets prevented the entry of mosquitoes, but not of sand flies.

“Sometimes, even if we try to use a net, it will allow the sand fly to pass, but not the mosquito, since it is very small.”

Interview, VL patient.

### Barriers to early healthcare seeking

The majority of participants across all respondent categories considered VL a very serious disease—both in terms of its detriment to physical health and financial consequences—with early care important. Nevertheless, few community members sought care at symptom onset, particularly among the MWs. Delays in seeking care ranged from several days to several months to several seasons.

Participants across all respondent categories reported that gaps in VL awareness inhibited early healthcare-seeking, with MWs coming from non-VL endemic areas particularly at risk. Sick individuals (both residents and non-residents alike) often failed to recognize VL. They confused their symptoms with those of other diseases—especially malaria—and underappreciated the seriousness of their situation. Many hoped for self-resolution of their illness or self-diagnosed and self-medicated for other diseases in lieu of seeking care at a health facility.

“The workers do not go to the health facility as soon as they feel the signs and symptoms of the disease because they assume that the disease is self-limiting.”

Interview, VL patient.

“…[MWs] travel with malaria drugs in their pocket. When they [MWs] get sick from VL they take malaria drugs and get some temporary relief”

Interview, Healthcare worker.

Socio-economic constraints constituted a major barrier to early healthcare-seeking. Sick individuals often lacked money to pay for healthcare services.

“The poor cannot afford 300 birr (approximately 11 euros) for a bed alone, and other expenses for food, syringes, needles and other services. If a poor person is asked to buy all those things, the day will be dark for him.”

FGD, Caretaker of VL patient.

While some local residents benefited from a health insurance plan that helped mitigate costs by reducing healthcare expenditures in exchange for an annual fee, MWs coming from outside the area were not eligible for the plan. Policy establishes the provision of social services in the form of fee waivers, however no patients interviewed (neither MWs, MRs, nor residents) mentioned having received such aid. One healthy MW interviewed even stated that this aid was not given to MWs.

“In the health centres, there are free health services to those who cannot pay the cost of the health services, even in the governmental health facilities. This free health service is not given to the MWs. So, it would be good if this [free health service] also included the MWs.”

FGD, MW.

Binding contracts and fear of loss of income also contributed to delayed healthcare-seeking. MWs and MRs relied on the income they earned during the agricultural season for subsistence for themselves and their families. They were highly dependent on the farm owners, who paid them upon completion of their work. Few farm owners paid workers who fell ill for partial work completion, or allowed workers to seek care and return later to complete their work. Accordingly, any break in agricultural activities for VL healthcare-seeking potentially resulted in major financial losses, both in terms of direct costs (i.e., healthcare fees) and indirect costs (i.e., loss of income during the month-long VL treatment).

“I am unfortunately sick right now. If I want to go to a health institution, he [the farm manager] will claim the unfinished contract. The manager wants to see all activities done. He does not want to allow me to go, even if my friends insist that he let me leave and promise to cover my side. He refuses it.”

Interview, MW.

“We farm owners don’t pay them unless they finish their contracts. This is usual and taken as tradition among the farm owners. In such cases, we request a MW to replace his position with someone else or to finish by himself. But, this is something wrong. This is because sometimes, MWs report and act as if they were sick. They do such things to escape from their work as it is not easy, I mean what they anticipated is different from the reality at the farm. Of course and in reality, it looks fair if I pay him for the number of days he worked for me, and I hope I will do so in the future.”

Interview, Community leader.

Social support, particularly decision-making systems within the family and community for choosing to pay for or seek external care, strongly influenced healthcare-seeking behavior. The caretaker, rather than the sick individual, was responsible for making decisions on care seeking and ensuring the quality of the care received. As caretakers are often family members, and MWs and MRs were usually far from their families, among the mobile population, the responsibility for decision-making for seeking care shifted to the sick MW/MR themselves.

“…most of the time, the decision depends on the caretakers, not on the patients, whether or not to go to the health institutions when they are sick with VL. But sometimes the patients can also say ‘please take me to the health institutions,’ but culturally it is the caretakers who carry the responsibility.”

Interview, Healthcare worker.

Community members reported that peers—especially those of MWs and MRs—also played an important role in decision-making and ongoing support, including helping the MW/MR to the health facility and taking over work responsibilities (as permitted by the farm owner).

“My friend advised me to go to [X] health centre and I went and they said it is typhoid fever from which I am suffering. Then a day after, I started working and I could not get better health and still they [my friends] told me to go to the hospital. They [my friends] advised me not to come back [to work] until I was in better health. I am here [at the hospital] because of them and it [my illness] was diagnosed as VL. I am here [at the hospital] and I have a better health due to my friends.”

Interview, VL patient.

Family and community members, especially those with a previous history of VL and therefore knowledge of VL, were influential in motivating sick individuals to seek healthcare.

“I took my brother here [health facility] because there is one herder, a friend of my brother, and he had VL two years ago. He got treatment and was cured. He asked my brother what his symptoms were and then he told us that it is VL. So we have to take him to a health facility.”

Interview, Caretaker of VL patient.

Family and community members also provided financial support. The charity of the community, including contributions from religious institutions, was important for some sick individuals (although this was reported by community leaders and MRs, but not by MWs themselves).

“If someone gets VL, all family members and relatives collaborate and help each other. However, if the family members cannot afford the cost of diagnosis and treatment, the community groups help the family.”

FGD, MR.

“If they do not have relatives and money, they go to their respective religious institutions (mosque/church).”

Interview, Community leader.

In addition, gaps in legal protections contributed to delayed healthcare-seeking. The law obliges farm owners to provide healthcare services, food, and water to their workers under contract. However, farm owners failed to meet their legal responsibilities—sometimes with government impunity—and often directly interfered with early healthcare-seeking. Furthermore, MWs did not benefit from sick leave.

“Even though the law says so, in most of the camps, there is no health service, no house for sleeping, and no safe food or water supply. Meaning, the workers, including the permanent employees, will not get these services. It is a very challenging life.”

FGD, MW.

Not all reports of farm owners by MWs were negative, however. Some farm owners provided advance payments to their workers for healthcare-seeking or took MWs to health facilities themselves. Yet this generally occurred when the MW had a special relationship with the farm owner (either a direct family tie or having worked for a long time with the farm owner) or to protect the farm owner or manager from problems with the government. Importantly, these interventions facilitated healthcare-seeking, but they still generally occurred late, once the health of the MW had already significantly deteriorated.

“…the farm owner will allow him to go…Since the MW stays with the farm owner for some time, he will give him [monetary support].”

Interview, Community leader.

### Healthcare-seeking and access to diagnosis

Unrelenting, increasingly severe illness motivated individuals to seek healthcare eventually.

“They [MWs] go to the health facility for VL diagnosis and treatment after their illness becomes very severe or worse.”

Interview, VL patient.

In addition to seeking relief from their suffering, healthcare seeking at this point represented, for many, awareness that their unresolved, worsening condition was not another common disease, but rather VL, for which they deemed healthcare necessary (i.e., it would not resolve on its own and they were unable to continue working).

Most community members and leaders considered hospitals as the best place for VL treatment, but prior to diagnosis and treatment, almost all sick individuals consulted other providers. Not a single MW reported the availability of HC services on farms.

Numerous factors influenced decisions regarding where to seek care. Sick individuals usually sought care at the health facility the shortest distance from their residence (local residents) or work location (MWs/MRs), or the one in closest proximity to their families to ensure having a caregiver. While most sick individuals started at the primary healthcare level, some reported that for more severe illnesses, they preferred seeking care directly from a higher-level health institution. Public health facilities were preferred due to their lower cost of care; however, individuals with access to financial resources visited private health facilities—considered as more efficient and respectful. Most community members and leaders, including traditional healers themselves, did not discount traditional medicine as a whole, but asserted that traditional medicine was not useful against VL. Visits to traditional healers were motivated by a lack of recognition of the signs and symptoms of VL by the sick individual and/or frustration with the failure of the modern health system to identify their illness.

Access to VL diagnosis consistently required multiple visits to health facilities. Sick individuals made as many as 10 repeat visits to the same health center for the same illness episode.

“If patients are not seriously ill, the health professional does not perform the VL test or will not refer to the place where the test is found. So, the health professionals cause a lot of damage to the patients. They wait until VL patients become complicated. The patients visit many times before receiving a diagnosis. The health center will not refer on time, [even though] you are telling them all the signs and symptoms of VL. They will not send you to the doctor delegated to diagnose VL. You will see the doctor after you develop different complications. So, the primary obstacle is the health centres themselves.”

FGD, MW.

Below, we present an analysis of the typical trajectory through the different healthcare modalities; this analysis is representative of VL patients and caregivers of VL patients in the study as they consistently reported their shared experience with these healthcare-seeking steps.

#### Analysis of the different healthcare-seeking steps consistently experienced by VL patients and caregivers of VL patients in the study prior to diagnosis of VL

Initially, sick individuals sought care from a private pharmacy, where they received paracetamol. As their condition worsened, they next went to the public health center, receiving malaria treatment. Their health did not improve and time passed. They returned to the public health center where, rather than being referred to a hospital, they were prescribed typhoid treatment, to no avail. Frustrated by the lack of diagnosis as well as their persistent and increasingly severe symptoms, some, but not all participants next went to a traditional healer for their still unknown disease. Depending on the type of traditional healer, they received either smoking of leaves or holy water. Given the higher fees associated with private versus public health care, only participants with access to sufficient financial resources went to a private health clinic next. There, patients were either finally referred to a higher health institution given suspicion of VL or once again received an incorrect diagnosis. After these significant delays, most patients were now severely ill. Thanks to social support or a healthcare professional referral, participants finally went to the hospital, where a diagnosis of VL was established.

The long and arduous journey in search of a diagnosis was costly for sick individuals. While VL diagnosis and treatment are free in Ethiopia, with each misdiagnosis and delay, patients paid for ineffective treatments and endured additional and increasingly severe symptoms and hardships, or even death, prior to diagnosis.

“We know medical care for VL is free of charge. We have already the information and even we got a kind of rumor. But, you don’t get free VL medical care starting from the very beginning to the very end…I myself spent a lot of money (I finished the money I had collected by different activities) and even some community members have contributed a lot in monetary terms to me…I really was exposed to a big expense.”

FGD, VL patient.

### Access to treatment

While access to diagnosis represented a major obstacle, access to treatment was much better.

“The process that comes after identifying the disease is free of problems. The problem lies during the process of disease identification.”

FGD, VL patient.

Once diagnosed, most patients reported rapidly starting treatment.

“They admitted me immediately [at Kahsay Abera hospital] and started medication on the next day.”

VL patient.

Furthermore, community members and leaders expressed a high level of confidence in modern VL treatment.

“All of them were cured after the thirty-day treatment given by injection.”

Interview, MW.

This confidence extended even to community members and leaders who mentioned patients dying after receiving injections to treat VL. Almost all of these participants stated that this did not make them question the treatment; rather, they reported that the patient initiated treatment too late, after their condition had severely deteriorated.

“My friend’s son was attacked by VL. When we took him to health facilities located here in Maykadra, they told us he had malaria, fever, and the like and he was treated for all identified diseases. Despite all the treatment, his condition was deteriorating. When we took him to the Abdurafi center, he died after two injections for VL. This is the problem of the residence of this area. You cannot get immediate treatment to the disease that you have.”

Interview, Community leader.

While the length of treatment and pain of injection were extremely burdensome, almost all patients adhered strictly to VL treatment.

“…this treatment is my life.”

Interview, VL patient.

### Burden of VL on the healthcare system

VL created a heavy financial and workload burden on the public healthcare system. Shortages of supplies and healthcare staff, allocated to health facilities based on the number of residents living in the community, were experienced during the massive influx of MWs.

“Therefore, this health center has served beyond its capacity due to the presence of too many daily workers. This led to a shortage of pharmaceutical drugs and other supplies. The supplies allocated for the health center are based on the number of residents in the community, but they treated many daily workers beyond the community residents.”

Interview, Political authority.

Low work attendance at the public health institutions by physicians busy in their private practice exacerbated understaffing. Furthermore, inadequate reporting within the healthcare system contributed to supply ruptures.

“…problem is not appropriately filling the report and request form. Therefore, there is a shortage of supplies.”

Interview, Healthcare worker.

Between health facilities, referrals, the flow of information, and feedback about patients functioned suboptimally.

“We do not have almost any follow up system for VL cases in our health center. In the previous times, we were registering all suspected VL cases in our outpatient department. We even strictly monitored the outcome of VL suspected cases referred to the hospital. But now, we do not know the exact number of suspected and confirmed VL cases due to the shortage of diagnostic supplies and follow-up system.”

Interview, Healthcare worker.

In the community, health extension workers—individuals affiliated with a health post/satellite of the health center and responsible for conducting door-to-door health outreach activities in their district [28]—lacked a formal referral form.

“We [health extension workers] do not have a referral form. We have for example a referral form for tuberculosis. If we suspect tuberculosis, we write a referral form immediately and we receive the feedback based on the referral. But in case of VL, it is verbal. There is no formal referral from health extension workers.”

Interview, Healthcare worker.

Furthermore, without ambulances, in the event of referrals, patients or their caregivers bore the responsibility of getting from one health facility to another.

Many participants, across all respondent categories—including healthcare workers themselves, reported that healthcare workers, from the lowest to highest cadres, were not adequately aware of or prepared for VL. Factors influencing knowledge and preparedness included the healthcare facility level, length of service in a VL endemic area, education attained, training received, and prevalence of VL (e.g., the healthcare worker’s exposure to patients with VL). The most capable healthcare workers included hospital staff, as well as those with a longer duration of service, superior level of education, ample training, and significant experience working in a highly VL-endemic area.

In accordance with national policy, diagnostic test kits and treatment were available at government hospitals. Despite some reports of bed shortages, as well as test kit, laboratory reagent, and drug supply ruptures, overall most participants, across all respondent categories, considered diagnosis and treatment of VL effective at the hospital level.

“…our hospitals, the probability of misdiagnosis/underdiagnosis is low.”

Interview, Healthcare worker.

“In Humera [Kahsay Abera Humera Hospital], the doctors are very cooperative and caring.”

FGD, Caretaker of VL patient.

However, the lack of VL diagnostic test kits at public health centres, as well as from private health facilities, constituted a major impediment to preparedness. This may be attributed to: 1.) The difficulty of diagnosing VL based on clinical signs and symptoms, and 2.) The resulting lack of prioritization and focus given to VL as the result of the inability to effectively diagnose it at the health centre.

“In the primary health centres, VL is commonly missed. But since most of the health centres diagnose using clinical signs and symptoms, there is high probability to miss the disease. Even if we suspect VL at the beginning, we do not refer the patient directly to higher health institutions. We choose to treat him as a non-VL patient for the other diseases that have some similarities with VL. Many VL patients are missed or not diagnosed due to the shortage of diagnostic test materials.”

Interview, Healthcare worker.

“Due to the unavailability of the diagnostic kit, we do not pay attention.”

FGD, Healthcare worker.

## Discussion

Bates et al. [29] describe vulnerability in terms of the factors that contribute to the unequitable impact of disease between different groups and individuals. Mobile populations often constitute a vulnerable group, at disproportionate risk of disease compared to non-mobile groups due to differences in social, economic, biological, environmental, and institutional factors [29]. Population movement is a major determinant of global public health and an important driver of infectious disease transmission [30]. Previous research in Ethiopia has shown an increased risk of malaria transmission among mobile workers linked to agricultural-related activities and mobility [23]. Similarly, the role of mobility in the risk of HIV infection is well documented [31–34]; a study [34] in Ethiopia found a higher risk of HIV infection among mobile workers from rural areas associated with temporary employment in an urban setting. In our study, numerous factors associated with vulnerability to VL [9,11,16] characterized MWs and MRs, who faced significant barriers to access to VL diagnosis and care. MWs had little awareness about VL. They had low income and many labor constraints. They depended on farm owners for their income and were under pressure to make as much money as possible. When falling ill, the decision to seek care required them to abandon their work and typically resulted in a total loss of income. MRs were also vulnerable given their similar risk for labor constraints and/or insufficient familial support linked to their transitory behavior and lower socioeconomic class. Addressing these barriers is essential to mitigate VL transmission and improve health.

The most common VL prevention method suggested by participants was the use of bed-nets, with residents reported to use them the most. In Uganda, Kolaczinkski et al.[17] found that among pastoralists, those who understood the severity and potential impact of VL were more likely to use a bed-net. Thus, in our study population, greater reported knowledge of VL among residents, compared to MWs, could explain their higher use of this protective measure. However, exclusion of MWs from bed-net distribution in Humera, given their lack of local residence, could also explain their limited reported usage. MWs needed either to carry a bed-net with them from home (assuming that they were from a malaria-endemic zone and thus a potential recipient of a government-issued, free bed-net) or purchase a bed-net, the price of which represented another barrier, similar to findings from other studies [35,36]. Lack of access to bed-nets has been previously reported among MWs; Schicker et al. [23] found that only 12% of MWs in the northern Amhara Region obtained access to a bed-net while engaged in agricultural activities in the area. Importantly, reasons for non-use of bednets were not limited to issues of access, but also to a lack of acceptability (e.g., bed-nets being uncomfortable for use during the high temperatures of the hot season, poorly adapted for outdoor use, and of questionable efficacy against VL given their mesh size). These factors suggest that while bed-net distribution to MWs might be beneficial, it would likely be insufficient on its own to encourage bed-net usage as a prevention method for VL.

Most participants talked about the importance of early healthcare-seeking behavior, however, similar to findings from other studies [17,23,37,38], few MWs and MRs sought care at the onset of symptoms. Once care was sought, participants recounted multiple visits to many different types of healthcare providers unable to diagnose and treat their illness. Anoopa et al. reported a similar finding in Bangladesh, where patients visited a median of six different healthcare providers prior to VL diagnosis [39]. The national health protocol instructs providers to consider VL among patients with more than two weeks of fever who are not responding to antimalarial medications [40]. In accordance with national policy, diagnostic test kits and treatment for VL are primarily available only at hospitals, necessitating referral to a higher health institution for VL assessment and care. Yet providers at public health centers repeatedly did not refer VL suspects to the hospital for evaluation. Instead, they treated patients for other illnesses for which medications were available at the health centres. Possible explanations for this include gaps in healthcare worker knowledge and/or reticence of providers to refer patients to a higher level of care prior to exhausting all possible options at the primary healthcare level. The repeat visits to health centres obliged patients to pay for, and physically support, treatments for diseases they did not have. Sick individuals suffered significant health consequences and financial losses prior to eventual diagnosis and treatment at hospitals. Paintain et al. found similar results in a cross-sectional survey of VL patients (approximately half of whom were MWs) treated in VL endemic areas in Ethiopia in 2016; the total financial costs for patients exceeded 31.4% of the annual household expenditure and the reported loss of earning for patients and caretakers resulted in an economic cost of illness nearly twice the financial cost [38].

### Validity and possible limitations of the study

Our study population included fewer MWs from the highlands than expected. This could have been due to the State of Emergency declared by the Ethiopian government on 09 October 2016 that may have resulted in fewer MWs coming from the highlands and other regions of the country to the lowlands of Western Tigray for agricultural work. Additionally, the State of Emergency delayed the commencement of the study, which may have contributed to the presence of fewer MWs in the area (i.e., MWs who came for agricultural work had already returned home when the study started.). As MWs from the highlands would be expected to be more vulnerable to VL and less familiar with VL than those from the lowlands, our study results may underrepresent the extent of VL vulnerability of MWs and overestimate the extent of VL knowledge of MWs. Importantly, our study participants included not only MWs, but also community leaders, healthcare workers, mobile residents, current/previous VL patients and caretakers of VL patients, all of whom who spoke not only of their own experiences, but also of the experiences of others.

### Generalizability of the study

The results of our study may not be entirely generalizable to populations at risk for VL, though importantly, this was not an objective of the research, given its qualitative design. Rather, our research aimed to explore in depth the opinions, beliefs, and feelings of the study population’s needs as they relate to VL diagnosis and treatment. Qualitative methods are increasingly used for health research given their suitability for better understanding peoples’ attitudes, behavior and decisions [25].

### Strengths, clinical relevance, and recommendations of the study

To our knowledge, our study is the first in Ethiopia to use qualitative methods to assess barriers to VL diagnosis and care among mobile seasonal workers. Accordingly, our results have important public health and policy implications. Specifically, our results may help guide the development of strategies to increase access to VL diagnosis, allowing for earlier treatment and better prognosis for VL patients, and reducing the potential health disparities and the overall burden of VL. Our study findings support the following recommendations:

#### Recommendations for the health system

- Decentralization of rapid diagnostic test kits to the primary health care level
  - With strict follow-up by the Tigray Regional Health Bureau and district administration offices
  - Including mentoring healthcare workers on correct use of the test kits
- On-going training and mentorship of all cadres of healthcare workers on VL
- Monitoring and evaluation of healthcare service delivery at all levels (from health posts up to the general hospitals, and including private health facilities)
  - Improved referral systems with a specific referral form for VL
  - Flow of patient information between health facilities
  - Reporting of VL among all health administration levels
- Healthcare service delivery at or near the farms (e.g., mobile clinics, mobile healthcare workers deployed for the agricultural season), including transportation services to provide a means of travel to/from (VL) treatment facilities to the farmlands
- Periodic adaptation of the workforce and health care supplies in relation to the changing population size

#### Recommendations for the community

- Practical health education on VL
  - Focused on when to suspect VL and where to go for VL diagnosis and care, rather than merely transmission and prevention
    - Emphasizing the importance of seeking treatment early
    - Mentioning specific hospitals in highlands providing VL care for highlanders falling ill upon their return home
  - Community-based delivery of health education in addition to the dissemination of information at the health facilities
- Consideration of “community focal persons” for VL (similar to what is done for TB) or “VL ambassadors” (former VL patients) to help raise awareness

#### Recommendations for the farmlands

- Practical health education on VL
  - Designed specifically for MWs
  - Incorporated into existing health education sometimes provided on farms
  - Repeated regularly to cover new workers arriving
- Distribution of bed-nets to MWs

#### Policy recommendations

- Improved application of labour laws concerning the provision of healthcare services, clean water, safe food, and housing to MWs by farm owners
  - Via monitoring by the district social affairs office and district health office
  - With measures to increase compliance among farm owners
- Investment in the design and development of novel VL prevention mechanisms (such as bed-net-style tents with small mesh or other innovations) adapted for and accepted by MWs
- Research to develop a vaccine against VL
- Research to improve treatment modalities: Shorter courses of treatment and treatment in more acceptable forms (i.e., a tablet, rather than injectable, form)
- Reinforcement of the obligation of fair contracts by farm owners that allow for paid sick leave, payment of health care costs, salary advances, and temporary sick leave
- A health insurance scheme for MWs
- Better organization/political representation of MWs
- Free comprehensive healthcare beyond free treatment for VL patients (such as to cover the cost of admission, a bed, syringes, and transportation to/between health facilities)

### Conclusions

Contrary to what health policy for VL dictates in this endemic setting, access to diagnosis was very poor and access to treatment was, consequently, significantly delayed. The free provision of diagnostic test kits and treatment is a very positive step. At the same time, none of our participants reported access to VL diagnostic test kits at the health center level, which represented a significant barrier to care. Patients incurred high expenses prior to finally being correctly diagnosed with VL, paid many expenses supplemental to VL treatment, and suffered a total loss of income during their lengthy hospital stays. Delays in diagnosis contributed to increased severity of disease, which, at best, led to additional financial losses and at worst, resulted in death. Interventions tailored to the socio-economic and health needs of MWs and MRs, as well as to other persons suffering from VL, are needed to reduce health disparities and the burden of VL.

## Supporting information

S1 Table Characteristics of the study population participating in interviews. (PDF)

S2 Table Characteristics of the study population participating in FGDs. (PDF)

S1 Checklist (PDF)
