# Supplementary material for: Barriers to access to visceral leishmaniasis diagnosis and care among seasonal mobile workers in Western Tigray, Northern Ethiopia: A qualitative study
Source: PLoS Negl Trop Dis. 2018 Nov 8;12(11):e0006778. doi: 10.1371/journal.pntd.0006778 (PMC6224040; doi:10.1371/journal.pntd.0006778)
Supplement: S1 Table — (PDF) [file pntd.0006778.s001.pdf]

S1 Table. Characteristics of study population participating in interviews

Participant category	Participant occupation/subcategory	Date of session	Duration of session	Place of interview (District-Ward, location)	Participant address (District-Ward)
Mobile worker	Hired farmer	01/31/17	00:32	Kafta Humera-Maykadra	Kafta Humera-Adebay
Mobile worker	Hired farmer	01/31/17	00:40	Kafta Humera-Maykadra	Kafta Humera-Humera county
Mobile worker	Hired farmer	01/31/17	00:44	Kafta Humera-Maykadra	Kafta Humera-Maykadra
Mobile worker	Hired farmer	01/31/17	00:40	Kafta Humera-Maykadra	Kafta Humera-Maykadra
Mobile worker	Hired farmer	02/22/17	00:45	Kafta Humera-Adebay	Kafta Humera-Adebay
Mobile worker	Hired farmer	02/22/17	00:42	Kafta Humera-Humera, town	Hawzen-Mydalu
Caretaker of VL patient	Caretaker of mobile resident	02/17/17	00:53	Kafta Humera-Rawiyan	Kafta Humera-Rawiyan
Caretaker of VL patient	Caretaker of mobile resident	02/17/17	00:39	Kafta Humera-Baeker	Kafta Humera-Baeker
Caretaker of VL patient	Caretaker of mobile resident	02/04/17	00:46	Kafta Humera-Humera, hospital	Kafta Humera-Adebay
Caretaker of VL patient	Caretaker of resident	01/31/17	00:52	Kafta Humera-Adebay	Kafta Humera-Adebay
Caretaker of VL patient	Caretaker of resident	02/04/17	00:48	Kafta Humera-Humera, hospital	Kafta Humera-Maykadra
Caretaker of VL patient	Caretaker of resident	02/07/17	00:40	Kafta Humera-Adebay	Kafta Humera-Adebay
Caretaker of VL patient	Caretaker of resident	02/17/17	00:47	Kafta Humera-Baeker	Kafta Humera-Baeker
Caretaker of VL patient	Caretaker of resident	02/17/17	00:45	Kafta Humera-Baeker	Kafta Humera-Baeker
Caretaker of VL patient	Caretaker of resident	02/17/17	00:32	Kafta Humera-Baeker	Kafta Humera-Baeker
VL patient	Mobile resident: Hired farmer	02/20/17	00:51	Kafta Humera-Bereket	Kafta Humera-Bereket
VL patient	Mobile worker: Tractor driver	01/28/17	00:52	Kafta Humera-Humera, hospital	Sheraro-Ziban Gedena
VL patient	Mobile worker: Hired farmer, gold miner	01/28/17	00:44	Kafta Humera-Humera, hospital	Wolkayt-Welel
VL patient	Mobile worker: Hired farmer, herder	02/07/17	00:45	Kafta Humera-Adebay	Kafta Humera-Adebay
VL patient	Mobile worker: Hired farmer	02/20/17	00:46	Kafta Humera-Bereket	Kafta Humera-Bereket
VL patient	Mobile worker: Hired farmer	02/22/17	00:43	Kafta Humera-Adebay	Kafta Humera-Adebay
VL patient	Mobile worker: Herder	02/22/17	00:38	Kafta Humera-Humera, hospital	Gohola-04
VL patient	Resident: Student	01/28/17	00:42	Kafta Humera-Humera, hospital	Awira-Wolkayt
VL patient	Resident: Susistence-level farmer	02/18/17	00:45	Kafta Humera-Rawiyan	Kafta Humera-Rawiyan
VL patient	Resident: Subsistence-level farmer	02/22/17	00:42	Kafta Humera-Humera, hospital	Kafta Humera-Adebay
Community leader	Nun	01/31/17	00:33	Kafta Humera-Adebay	Kafta Humera-Adebay
Community leader	Priest	01/31/17	00:41	Kafta Humera-Adebay	Kafta Humera-Adebay
Community leader	Imam	02/04/17	00:43	Kafta Humera-Maykadra	Kafta Humera-Maykadra
Community leader	Political authority	02/07/17	01:55	Kafta Humera-Adebay, administration	Kafta Humera-Adebay
Community leader	Farm owner	02/07/17	00:42	Kafta Humera-Adebay	Kafta Humera-Adebay
Community leader	Farm/Camp manager	02/07/17	00:35	Kafta Humera, Goblel farm	Kafta Humera-Adebay
Community leader	Priest	02/11/17	00:44	Kafta Humera-Maykadra	Kafta Humera-Maykadra
Community leader	Political authority	02/11/17	00:54	Kafta Humera-Maykadra	Kafta Humera-Maykadra
Community leader	Farm owner, member women's orgranization	02/14/17	00:45	Kafta Humera-Adebay	Kafta Humera-Adebay
Community leader	Farm owner, member women's orgranization	02/14/17	00:45	Kafta Humera-Adebay	Kafta Humera-Adebay
Community leader	Civic authority	02/17/17	00:59	Kafta Humera-Humera, administration	Kafta Humera-Humera town
Healthcare worker	Nurse	01/28/17	00:58	Kafta Humera-Humera, hospital	Kafta Humera-Humera town
Healthcare worker	Pharmacist	01/28/17	00:28	Kafta Humera-Humera, hospital	Kafta Humera-Humera town
Healthcare worker	Nurse	01/28/17	01:00	Kafta Humera-Humera, hospital	Kafta Humera-Humera town
Healthcare worker	General practitioner	01/28/17	01:06	Kafta Humera-Humera, hospital	Kafta Humera-Humera town
Healthcare worker	Hospital authority	01/28/17	00:45	Kafta Humera-Humera, hospital	Kafta Humera-Humera town
Healthcare worker	Private practitioner	01/31/17	00:34	Kafta Humera-Adebay	Kafta Humera-Adebay
Healthcare worker	District health authority	02/04/17	01:00	Kafta Humera, District health office	Kafta Humera-Humera town
Healthcare worker	Health centre authority	02/07/17	00:42	Kafta Humera-Bereket	Kafta Humera-Bereket
Healthcare worker	Pharmacist (Private drug shop owner)	02/11/17	00:40	Kafta Humera-Maykadra	Kafta Humera-Maykadra
Healthcare worker	Private practitioner	02/10/17	00:42	Kafta Humera-Maykadra	Kafta Humera-Maykadra
Healthcare worker	Traditional healer, subsistence-level farmer	02/17/17	00:40	Kafta Humera-Humera, town	Kafta Humera-Humera town
Healthcare worker	Nurse	02/22/17	00:51	Kafta Humera-Humera, hospital	Kafta Humera-Humera town
Healthcare worker	Doctor	02/23/17	00:32	Kafta Humera-Humera, hospital	Kafta Humera-Humera town
Healthcare worker	Traditional healer, subsistence-level farmer	02/22/17	00:50	Kafta Humera-Maykadra	Kafta Humera-Maykadra
