# Supplementary material for: Barriers to access to visceral leishmaniasis diagnosis and care among seasonal mobile workers in Western Tigray, Northern Ethiopia: A qualitative study
Source: PLoS Negl Trop Dis. 2018 Nov 8;12(11):e0006778. doi: 10.1371/journal.pntd.0006778 (PMC6224040; doi:10.1371/journal.pntd.0006778)
Supplement: S2 Table — (PDF) [file pntd.0006778.s002.pdf]

S2 Table. Characteristics of study population participating in focus-group discussions

Participant category	Participant occupation/subcategory	Date of session	Duration of session	Place of interview (District-Ward, location)	N
Mobile workers	Hired farmers	02/05/17	01:20	Kafta Humera-Maykadra	10
Mobile workers	Hired farmers	02/14/17	01:47	Kafta Humera-Humera, town	9
Mobile residents	Wives of hired farmers	02/05/17	01:10	Kafta Humera-Maykadra	10
Caretakers of VL patients	Caretakers of residents	02/14/17	01:54	Kafta Humera-Adebay	7
Caretakers of VL patients	Caretakers of residents	02/20/17	01:07	Kafta Humera-Rawiyan	6
VL patients	Mobile residents: Hired farmers	02/14/17	01:45	Kafta Humera-Baeker	7
VL patients	Residents: 3 subsistence-level farmers, 2 students, 1 driver	02/11/17	01:53	Kafta Humera-Maykadra	6
VL patients	Residents: 1 security guard, 3 herders, 1 gold miner, 1 unspecified	02/14/17	01:55	Kafta Humera-Adebay	6
Healthcare workers	1 pharmacy technician, 5 nurses, 1 public health nurse, 2 health extension workers	02/07/17	01:39	Kafta Humera-Bereket	9
Healthcare workers	Heads of health centers	02/21/17	01:32	Kafta Humera-Humera, town	8
Healthcare workers	Health extension workers	02/20/17	01:13	Kafta Humera-Humera, hospital	9
